# The role of a urine dipstick in the diagnosis of the acute scrotum

**DOI:** 10.1002/bco2.138

**Published:** 2022-01-25

**Authors:** Sophie Tissot, Christopher Perrott, Richard Grills

**Affiliations:** ^1^ Department of Urological Surgery, Barwon Health University Hospital Geelong Geelong Australia; ^2^ Department of Surgery Deakin University Geelong Australia

**Keywords:** diagnosis, dipstick, haematuria, pyuria, scrotum, torsion

## Abstract

**Objective:**

To evaluate the role of a urine dipstick in the assessment of acute scrotal pain emergency department presentations.

**Methods:**

A single institution, prospective case series, from February 2020 to February 2021. All patients who received a bedside review by a urology doctor for acute scrotal pain were included. Urine dipstick results were pre‐defined as having had an impact on the emergency clinician's diagnosis if it showed pyuria and/or nitrituria and the final diagnosis was epididymitis‐orchitis or haematuria and the final diagnosis was ureterolithiasis.

**Results:**

139 patients presented to the emergency department with a complaint of acute scrotal pain. 85 (61%) were referred for bedside urology review. Median age of 17 years (P25 12 yrs, P75 31 yrs). 2.3% (*n* = 2) had proven testicular torsion, 28.5% (*n* = 24) had epididymitis‐orchitis and 8.2% (*n* = 7) had ureterolithiasis. 68 (80%) of patients received a primary diagnosis of testicular torsion by the emergency department clinician. Following review by a urology unit doctor, 14 proceeded to scrotal exploration for concern of testicular torsion. 7 patients were diagnosed with ureterolithiasis, all of whom had haematuria on their urine dipsticks (100%, 95% CI: 59–100%), 100% of these urine dipsticks were initiated by the urology unit doctor following bedside review. 22 patients were diagnosed with epididymitis‐orchitis. 8 of these had pyuria, nitrituria and/or haematuria on their urine dipstick (36%, 95% CI: 17–59%) and only one urine dipstick was completed prior to referral. 20.6% of patients perceived to have testicular torsion by the emergency department had a positive urine dipstick that aligned with their final alternative diagnosis (95% CI: 12–32%).

**Conclusion:**

A collection of clinical findings is required to diagnose the aetiology of acute scrotal pain. Information that can be easily, quickly, cheaply, and reliably collected, such as a urine dipstick, can assist in clinical decision making.

## 1 INTRODUCTION

Scrotal pain is a common emergency department presentation, with a spectrum of probable diagnoses ranging from a benign pathology requiring no treatment, to acute testicular torsion requiring urgent operative intervention.^1^ Haday and Reynard highlighted in their meta‐analysis the need for exploration of the testis within 6 h of pain, with a testicular viability of 98% within this time.^2^ The concern for missing the diagnosis of a testicular torsion and subsequently delaying intervention resulting in orchidectomy for an ischaemic testicle necessitates the hasty referral of patients for specialty review.

In addition to time pressures, acute scrotal pain is a complex clinical presentation for junior doctors to navigate. Menzies‐Wilson et al recently demonstrated, using the English National Health Service Trust data, that the individual doctors' experience was the greatest risk to misses of testicular torsion in 88% of cases.^3^ Due to the variability in clinical presentations, and the lack of an accurate, simple and timely diagnostic tool to rule out testicular torsion, patients are frequently referred to a urology unit doctor for specialist bedside assessment.

Differential diagnosis of torsion of the testicular appendage, epididymo‐orchitis or ureterolithiasis is also found in patients who present with acute scrotal pain. The presence of haematuria, pyuria and/or nitrituria in the setting of infection and the presence of haematuria in the setting of ureterolithiasis may assist in guiding towards these alternative diagnoses.

We aimed to quantify the role of urine dipsticks in the diagnosis of the aetiology of acute scrotal pain emergency department presentations and the impact it could have on the initial emergency department referral diagnosis if it were to be completed prior to contacting a urology unit doctor.

## 2 METHODS

A prospective case series of males presenting with acute scrotal pain to a single institution over a 12‐month period from the 3rd of February 2020 to the 31st of January 2021 was undertaken. Only patients who were referred to and reviewed at the bedside by a doctor working in the urology department were included. Ethic's approval was gained from the Barwon Health Ethics Department, Reference Number #20/181. Data were collected prospectively by the treating urology unit doctor and stored in a secure database.

Information collected included the emergency department doctors' provisional referral diagnosis, time of referral and if a urine dipstick had been completed prior. The time to urology bedside review, the urology doctors' assessment, investigation results and final diagnosis were also recorded. The final diagnosis was predefined as the diagnosis given by the urology unit doctor at the completion of their bedside assessment or the operative findings if the patient proceeded to a scrotal exploration.

A review of the urine dipstick results and their likely impact on the initial emergency department diagnosis was completed. The urine dipstick was predefined as having had an impact on the emergency clinicians' diagnosis if it showed haematuria, pyuria and/or nitrituria and the final diagnosis was epididymo‐orchitis or it showed haematuria and the final diagnosis was ureterolithiasis. Confidence intervals for positive dipstick proportions were computed using the Clopper–Pearson exact method.^4^ The association between positive urine dipstick and final diagnosis was tested using the chi‐square test.

The Victorian Emergency Department Dataset (VEDD) ICD‐10 codes allocated to patients at triage for their presenting complaint were used to collect the number of patients who presented to the emergency department with acute scrotal pain within the study period. Codes included were E299: Testicular Dysfunction, unspecified, N44: Torsion of testis, S302: Contusion of external genital organs, Q5390: Undescended testicle and N459: Orchitis, epididymitis and epididymo‐orchitis without abscess.

## 3 RESULTS

One hundred thirty‐nine patients presented within the research period to the emergency department with a complaint of acute scrotal pain according to the preselected VEDD ICD‐10 codes. Eighty‐five (61%) of these emergency department presentations were referred for bedside urology review in 79 different patients, and this was our study cohort.

The median age of patients presenting with acute scrotal pain was 17 years (IQR: 12–31 years). 2.3% (*n* = 2) had testicular torsion, 28.2% (*n* = 24) had epididymo‐orchitis, and 8.2% (*n* = 7) had ureterolithiasis (Table 1). A small number of patients presented with symptomatic varicoceles and hydroceles; four had testicular trauma with requiring surgical intervention for testicular rupture. A 5‐year‐old presented with an incarcerated inguinal hernia, and a 10‐year‐old presented with a first presentation of a painful undescended testicle.

**TABLE 1:** Characteristics of acute scrotal pain presentations by final diagnosis

	Torsion of testicle	Torsion of testicular appendix	Epididymo‐orchitis	Ureterolithiasis	Testicular trauma	Normal testis
Number	2 (2)	13 (15)	24 (28)	7 (8)	4 (5)	20 (24)
Age	31 (28,33)	11 (10,13)	19 (14,32)	33 (24,34)	16 (14,20)	17 (14,21)
Duration of pain	13 (8,19)	12 (2,48)	32 (5,72)	6 (3,18)	30 (19,45)	5 (3,21)
Acute onset of pain	1 (50)	10 (77)	9 (38)	5 (71)	1 (25)	14 (70)
Fever	0	0	1 (4)	0	0	0
LUTS	0	1 (8)	5 (21)	1 (14)	0	2 (10)
Vomiting	0	0	2 (8)	1 (14)	1 (25)	1 (5)
Abdominal pain	0	0	7 (29)	3 (43)	0	2 (10)
High testicle	1 (50)	2 (15)	2 (8)	2 (29)	0	2 (10)
Cremaster present	1 (50)	10 (77)	19 (79)	6 (86)	4 (100)	15 (75)
Erythema	1 (50)	0	5 (21)	0	0	0
Swelling	0	1 (8)	13 (54)	0	1 (25)	2 (10)
Positive urine dipstick	0	0	9 (37.5)	7 (100)	0	1 (5)
Imaging confirmed diagnosis	1 (50)^a^	0	18 (75)	6 (86)^b^	2 (50)	8 (40)
Raised inflammatory markers	0	0	6 (25)	1 (14)	1 (25)	1 (5)
Kidney injury	0	0	0	2 (29)	0	0

*Notes*: Data are reported as median (P25, P75) or *n* (%). Patients not presented in this table include symptomatic varicoceles or hydrocele, chronic pain, osteitis pubis, undescended testis and inguinal hernia due to their low numbers.

Abbreviation: LUTS, lower urinary tract symptoms.

^a^ External ultrasound.

^b^ Three ureterolithiasis diagnosed on CT KUB, two had unilateral hydronephrosis on US KUB, one had a renal calculi on CT, and one had imaging following resolution of stone but no stone seen.

Sixty‐eight patients who presented with testicular pain received a diagnosis of probable testicular torsion by the emergency department clinician. 44.1% of whom were reviewed by an emergency department consultant, the remainder of whom were reviewed by a senior emergency department registrar. Within this cohort, the median time from triage to urology unit referral was 55 min (IQR: 34–121 min). The median time from referral to urology unit review was 28 min (IQR: 15–45 min).

Fourteen were reviewed by the urology unit doctor and proceeded to scrotal exploration for concern for testicular torsion. Two of these patients had testicular torsion, three had normal testicles, four epididymo‐orchitis, and five had torsion of the testicular appendage.

**FIGURE 1:**
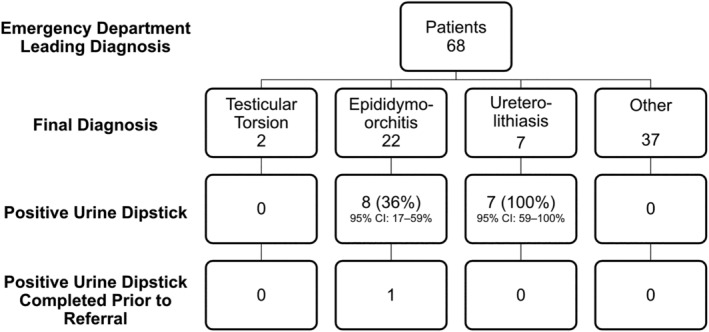
Urine dipstick in possible testicular torsion

Of the patients whom the urology unit doctor examined and deemed not to have testicular torsion, 32% were diagnosed with epididymo‐orchitis. Of these patients, eight had positive urine dipsticks with either haematuria, pyuria or nitrituria, and only one urine dipstick was completed prior to referral (Figure 1). Only one of these patients went on to grow *Escherichia coli* in their urine culture. A positive urine dipstick aligned with the alternative diagnosis in 8/22 epididymo‐orchitis cases (36%, 95% CI: 17%–59%).

Seven patients referred to a urology unit doctor with probable testicular torsion were diagnosed with ureterolithiasis. All of whom had positive urine dipsticks for haematuria (100%, 95% CI: 59%–100%), none of which were completed prior to urology unit referral (Figure 1). Two patients had CT confirmed <3.5‐mm vesicoureteric junction calculus, and one an intravesical calculus. Two patients had unilateral hydronephrosis seen on ultrasound of the kidneys, but no ureterolithiasis visualised. Two patients had CT KUB completed once pain had resolved, no ureteric or intravesical calculi were seen, but both had small renal calculi and a pain history indicative of renal colic.

Twenty (29.4%) of patients who were referred with possible testicular torsion had a urine dipstick completed prior to referral, and 20.6% of patients referred had a positive urine dipstick that aligned with their alternative diagnosis (95% CI: 12%–32%). Ninety per cent of these urine dipsticks were initiated by the urology unit doctor at the time of bedside review. A positive urine dipstick strongly associated with a diagnosis other than testicular torsion (chi‐squared 38.333, DF = 3, *p* < 0.001).

## 4 DISCUSSION

Patient care is at the forefront of all clinical interactions. A healthy working relationship between the emergency and urology departments is paramount to the management of patients with acute scrotal pain. A mutual respect for each other's clinical judgement and experience allows for optimisation of patient care.

The diagnosis of acute scrotal pain, in particular testicular torsion, cannot be reliably made based on a single investigation but instead a collection of clinical findings.^5^, ^6^ As such, any additional information that can be quickly and cheaply collected can assist in clinical decision making; a urine dipstick is an example of such an investigation. Timing of this urine dipstick should be prior to referral to a urology unit doctor, particularly if calling overnight or at least prior to their bedside review as long as no critical delays are encountered in gaining the dipstick results. Our study showed one in five patients perceived to have testicular torsion by the emergency department had a positive urine dipstick that aligned with their final clinical diagnosis of either epididymo‐orchitis or ureterolithiasis.

Within the literature, there is limited information on the role of urine dipstick in the diagnosis of acute scrotal pain. Frohlich et al did a prospective analysis of 258 paediatric patients less than 18 years of age who presented with acute scrotal pain. Six of the 19 patients with testicular torsion had a urinalysis completed, and all were negative for blood or leukocytes.^6^ Roth et al conducted a retrospective study of 440 consecutive patients referred with scrotal pain to a university hospital in Switzerland. Of their cohort, the leading cause of acute scrotal pain was genital/paragenital infection accounting for 58.7% of presentations. 9.5% had testicular torsion and 2.5% had ureterolithiasis. In multivariate regression analysis, a leukocyte count of >4 per field in the urine was an independent and statistically significant predictor of genital/paragenital infection.^5^ Interestingly within this cohort, two (4.8%) of the 42 patients with testicular torsion had both elevated leukocytes in blood and urine, and the paper makes comment that this may have been due to concurrent infection. However, it is important to highlight that a positive urine dipstick does not always support a lack of testicular torsion and bedside assessment and clinical judgement remain paramount in the management of this patient cohort.

Limitations of our study include the small sample size, in particular the low number of patients with testicular torsion in our cohort. As a single‐institution study, it represents only one emergency and one urology department's management of patients with acute scrotal pain.

Despite these limitations, our data currently justify the routine use of urine dipstick in the workup of acute scrotal pain. Clinical integration into diagnostic algorithms such as the TWIST score^7^ for the workup of testicular ischemia may occur into the future, with a positive urine dipstick result equating to a negative score towards a testicular torsion diagnosis. Acknowledging that its integration would need to occur with the overarching principle that clinical judgement in each clinical scenario remains paramount.

## 5 CONCLUSION

A urine dipstick is a contributing adjunct in the workup of patients who present to the emergency department with acute scrotal pain. The result of the urine dipstick should be used in conjunction with other clinical findings and if concern remains that the patient has testicular torsion irrespective of the dipstick result the clinician should proceed to scrotal exploration.

## CONFLICT OF INTEREST

The authors declare there is no conflict of interest.
